# Little noticed, but very important: The role of breeding sites formed by bamboos in maintaining the diversity of mosquitoes (Diptera: Culicidae) in the Atlantic Forest biome

**DOI:** 10.1371/journal.pone.0273774

**Published:** 2022-09-06

**Authors:** Gerson Azulim Müller, Cecilia Ferreira de Mello, Anderson S. Bueno, Wellington Thadeu de Alcantara Azevedo, Jeronimo Alencar

**Affiliations:** 1 Instituto Federal de Educação, Ciência e Tecnologia Farroupilha, Panambi, RS, Brazil; 2 Laboratório de Diptera, Instituto Oswaldo Cruz (Fiocruz), Rio de Janeiro, RJ, Brazil; 3 Programa de Pós-Graduação em Biologia Animal, Instituto de Biologia (UFRRJ), Universidade Federal Rural do Rio de Janeiro, Seropédica, RJ, Brazil; 4 Instituto Federal de Educação, Ciência e Tecnologia Farroupilha, Júlio de Castilhos, RS, Brazil; Virginia Polytechnic Institute and State University, UNITED STATES

## Abstract

This study investigated the composition of mosquito species in different kinds of breeding sites in a tropical forest remnant of the Atlantic Forest and identified species of public health concern therein. Collections of immature forms of mosquitoes were carried out monthly at the Poço das Antas Biological Reserve in southeastern Brazil, between June 2014 and June 2015. Samples were collected from four types of breeding sites: bamboos, bromeliads, puddles, and a lake. A total of 1,182 specimens of mosquitoes belonging to 28 species and 13 genera were collected. Three species, *Ad*. *squamipennis*, *An*. *neglectus*, and *Wy*. *arthrostigma* represented 64.8% of the captured specimens. Only three species were found in more than one type of breeding site: *Ps*. *ferox*, *An*. *triannulatus*, and *Tx*. *trichopygus*. Two species of public health concern were found breeding in bamboo (*Ae*. *aegypti* and *Ae*. *albopictus*) and one in the lake (*An*. *darlingi*). Bamboo had the highest species richness, Shannon diversity, abundance of individuals and number of dominant species of all breeding sites. Similar Simpson diversity was obtained for bamboo and bromeliads, with higher values than those obtained for puddles and the lake. The significance of the four breeding sites, especially bamboos, is discussed in the context of controlling populations of sylvatic species of mosquitoes in Atlantic Forest areas.

## Introduction

In recent decades, the recurrent emergence of infectious diseases caused by zoonotic agents, especially those transmitted by mosquitoes, has caused severe public health problems, especially in subtropical and tropical countries like Brazil. Epidemic diseases such as chikungunya, dengue, yellow fever, malaria, and zika, which cause the illness and deaths of millions of people, are a challenge for control programs by health authorities [1–3].

Entomological surveillance practices and vector control are generally employed together to predict and control urban diseases such as dengue [4]. However, few surveillance and control strategies include natural hedges in sylvatic environments [5]. To extend these programs, it is first necessary to know which species of mosquitoes occur in these areas and understand the role of potential breeding sites in controlling this insect fauna.

The Atlantic Forest extends across the south, southeast, and northeast regions of Brazil. This biome has a forest cover of 32 million hectares, corresponding to 29% of its original area, and is one of the global biodiversity hotspots [6]. Despite its severe degree of forest loss and fragmentation, the Atlantic Forest harbors approximately 100 mosquito species [7–9]. Several of these species are associated with the transmission of pathogens that cause human diseases, like *Anopheles cruzii* [10] and *Haemagogus leucocelaenus* [11].

The number of mosquito species in a given area is associated, among other factors, with the number and types of breeding sites available for these insects [12]. Hence, immature forms of mosquitoes can be found in various aquatic habitats if they contain standing water. These breeding sites, classified as permanent and semi-permanent or transient, are formed by the accumulation of water (e.g., lakes) or natural and artificial containers [13]. The breeding sites formed from water accumulation in plants or plant parts are called phytotelmata. These breeding sites can be formed in bromeliads, bamboo internodes, tree hollows, fruit peels, plant bracts, and even when water accumulates in carnivorous plants [14].

Generally, most mosquito species of the Aedeomyiini, Aedini, and Mansoniini tribes occur in breeding sites formed by water accumulation in the soil. However, Sabethini and Toxorhynchitini mosquitoes are mainly found in breeding sites formed by natural containers such as phytotelmata. Some species, like *Aedes terrens*, are more generalists, using breeding sites of different kinds [15, 16], while others, such as *Sabethes aurescens*, are more specific [17].

Most studies on mosquito breeding sites in the Atlantic Forest focus on bromeliads (e.g., Chaves et al. [18]) or bamboos (e.g., Müller et al. [19]). However, few have compared different types of breeding sites in the same area. Thus, the present study aimed to compare the composition of the mosquito community found in several types of breeding sites in an tropical forest remnant of the Atlantic Forest and identify areas where species of public health concern occur.

## Material and methods

### Ethics statement

The research project was conducted at the Poço das Antas Biological Reserve with the authorization for collecting zoological material No. 44333–1, issued on 05/06/2014 by the Department of Environment and Agriculture. The permanent license, number 34911–1, for collecting, capturing, and transporting zoological material was granted by SISBIO on 06/14/2012.

### Study area

**Fig 1:**
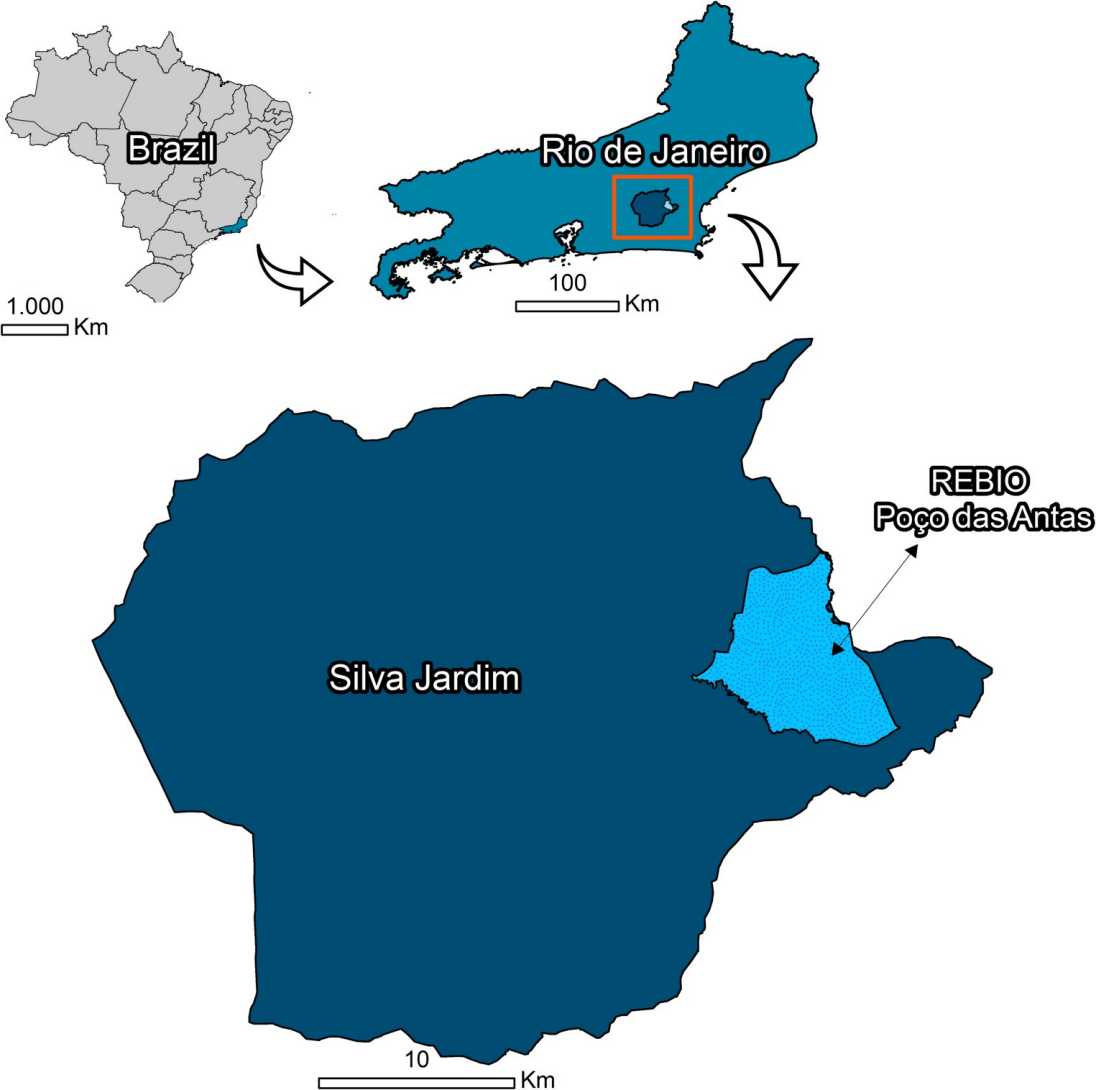
Sampling site in the Poço das Antas Biological Reserve in southeastern Brazil. Maps were prepared using QGIS 3.14.16 software and edited in Adobe Photoshop CS5 and Corel Draw X5. Reprinted from QGIS 3.14.16, a program under a CC BY license, with permission from Jeronimo Alencar—Fiocruz, original copyright 2021.

The collections were carried out at the Poço das Antas Biological Reserve (PABioR), located in Silva Jardim, State of Rio de Janeiro, Brazil (22°30’–22°33’ S, 42°15’–42°19’ W; Fig 1). The region’s climate is hot and humid with a rainy season in the summer and high average annual temperatures, without a pronounced winter. According to Takizawa [20] and Cunha [21], the average annual temperature varies from 21.4°C to 24.30°C.

PABioR is a strictly protected area (IUCN category Ia) spanning 5,065 ha, with vegetation cover primarily consisting of Lowland Ombrophilous Dense Forest, which can be divided into flooded and non-flooded forests. The relief of the reserve is characterized by low hills [22]. This terrain includes a mosaic of forest fragments with different levels of preservation that are often connected through corridors of native forest.

### Collections and laboratory procedures

Immature mosquitoes were collected from four types of breeding sites: bamboos, bromeliads, puddles, and a lake (S1 Fig). The first three types of breeding sites were located within a forest fragment near the PABioR head office, while the lake–the reservoir of an artificial dam–was in an area adjacent to the same forest fragment.

The studied forest fragment is crossed by trails and a small road used to travel through the Biological Reserve. It has dense vegetation, with a forest canopy of up to 6 m high shading an irregular understory. Native and typical plants of the Atlantic Forest were observed in the fragment, belonging to families including Fabaceae, Bignoniaceae, and Myrtaceae. The area surrounding the lake was directly exposed to sunlight and covered by shrubs and herbaceous species.

A total of 13 field trips of two days each were carried out monthly between June 2014 and June 2015. In each field trip, 11 bromeliads, eight bamboos (five internodes per plant), 21 puddles, and a lake were convenience sampled (i.e., non-probabilistic sampling).

Water samples from bromeliads were placed in plastic trays using a manual sucker, and immature mosquitoes were collected from the trays as described by Müller and Marcondes [23]. Bamboo samples were obtained from five internodes per plant, on average, by using an electric drill as described by Marcondes and Mafra [24]. After 30 days, the drilled internodes were cut, and their water content was analyzed in plastic trays to collect immature mosquitoes. Meanwhile, water samples from puddles and the lake were obtained using dippers, as described by Forattini [13], and processed in trays as previously described. Larvae and pupae samples were transported in 250 mL plastic bags (Whirl-Pak®) and labeled with information on the location, date, and type of breeding site where the collection was made.

In the laboratory, immature mosquitoes were sorted and transferred to small individual vessels with water from the breeding site in which they were collected. These vessels were periodically topped up with distilled water. Predatory larvae, including those of the genus *Toxorhynchites*, were fed with *Aedes aegypti* larvae from a colony maintained in the laboratory. The immatures were reared to continue their life cycle, and exuviae and dead larvae were preserved in 70° GL ethanol for later identification. The pupae were transferred to small sludges, in which they stayed until the adult stage.

Specimen identification was carried out by direct observation of morphological traits under an optical microscope. Immatures were fixed on slides with coverslips, while a stereomicroscope was used for adults, which were mounted on paper triangles affixed to entomological pins. The morphological traits observed were consulted in dichotomous keys and descriptions from Lane [25], Consoli and Oliveira [26], Forattini [13] and Stein et al. [27]. In addition, identifications were conducted by comparing the collected specimens with ones kept in the reference collection of the Oswaldo Cruz Institute (Fiocruz). The collected and analyzed specimens were listed in the Entomological Collection of the Instituto Oswaldo Cruz under the title “Coleção Mata Atlântica.” Genera and subgenera were abbreviated following Reinert [28].

### Data analysis

To assess the effect of the type of breeding site on species diversity, we employed the framework of Hill numbers [29] using individual-based species accumulation curves standardized by the number of individuals captured. Accordingly, three diversity indices were used: (i) species richness, (ii) the exponential of Shannon’s entropy index (hereafter, Shannon diversity), and (iii) the inverse of Simpson’s concentration index (hereafter, Simpson diversity), which can be interpreted as (i) the number of species, (ii) the number of typical species, and (iii) the number of very abundant species in the community [30]. These three indices share a common set of intuitive mathematical properties and are expressed as the ‘effective number of species’–i.e., the number of equally abundant species that would be needed to give the same value of a diversity measure [29]. Four species accumulation curves were generated by extrapolation to 827 individuals based on 1,000 bootstrap replications, one curve for each type of breeding site, with the effective number of species on the *y*-axis and the number of individuals captured on the *x*-axis. If the 95% confidence intervals of the species accumulation curves did not overlap, the difference in the effective number of species was considered significant.

Mosquito species were classified into dominant or subordinate. Accordingly, species with a relative abundance higher than 1/*S*, where *S* is the number of species, are considered dominant and subordinate otherwise. Graphs and analyses were performed in the R software [31] using the *iNEXT* package [32].

## Results

A total of 1,182 specimens of mosquitoes were collected, comprising 28 species, 13 genera, six tribes, and two subfamilies. The genus with the highest number of species was *Culex* (n = 6), followed by *Anopheles* (4), *Wyeomyia* and *Aedes* (3), *Limatus* and *Toxorhynchites* (2), and *Aedeomyia*, *Psorophora*, *Coquillettidia*, *Mansonia*, *Onirion*, *Sabethes* and *Trichoprosopon* (1). Three species, *Ad*. *squamipennis*, *Cx*. *neglectus* and *Wy*. *arthrostigma*, represented 64.8% of the total number of captured specimens (Table 1).

**Table 1:** Mosquito taxa collected from June 2014 to June 2015 in breeding sites located at the Poço das Antas Biological Reserve in southeastern Brazil.

Subfamily	Tribe	Species	N (%)
Anopheline		*Anopheles* (*Anopheles*) *maculipes* (Theobald, 1923)	2 (0.2)
		*Anopheles* (*Nyssorhynchus*) *albitarsis* Lynch-Arribalzaga, 1878	10 (0.8)
		*Anopheles* (*Nyssorhynchus*) *darlingi* Root, 1926	5 (0.4)
		*Anopheles* (*Nyssorhynchus*) *triannulatus* s.l. (Neiva & Pinto, 1922)	41 (3.5)
Culicinae	Aedeomyiini	*Aedeomyia* (*Aedeomyia*) *squamipennis* (Lynch Arribalzaga, 1878)	189 (15.9)
	Aedini	*Aedes* (*Ochlerotatus*) *serratus* (Theobald, 1901)	10 (0.8)
		*Aedes* (*Stegomyia*) *aegypti* (Linnaeus, 1762)	2 (0.2)
		*Aedes* (*Stegomyia*) *albopictus* (Skuse, 1894)	5 (0.4)
		*Psorophora* (*Janthinosoma*) *ferox* (von Humboldt, 1819)	66 (5.6)
	Culicini	*Culex* (*Melanoconion*) *pereyrai* Duret, 1967	10 (0.8)
		*Culex* (*Melanoconion*) sp.	5 (0.4)
		*Culex* (*Microculex*) *neglectus* Lutz, 1904	369 (31.2)
		*Culex* (*Microculex*) *pleuristriatus* Theobald, 1903	4 (0.4)
		*Culex* (*Microculex*) *reducens* Lane & Whitman, 1951	5 (0.4)
		*Culex* (*Microculex*) sp.	2 (0.2)
	Mansoniini	*Coquillettidia* (*Rhynchotaenia*) *venezuelensis* (Theobald, 1912)	2 (0.2)
		*Mansonia* (*Mansonia*) *titillans* (Walker, 1848)	2 (0.2)
	Sabethini	*Limatus durhamii* Theobald, 1901	3 (0.3)
		*Limatus pseudomethysticus* (Bonne-Wepster and Bonne, 1920)	2 (0.2)
		*Onirion personatum* (Lutz, 1904)	62 (5.2)
		*Sabethes* (*Peytonulus*) *identicus* Dyar and Knab, 1907	73 (6.2)
		*Trichoprosopon digitatum* (Rondani, 1848)	4 (0.4)
		*Wyeomyia* (*Miamyia*) *oblita* (Lutz, 1904)	70 (5.9)
		*Wyeomyia* (*Phoniomyia*) sp.	4 (0.4)
		*Wyeomyia* (*Wyeomyia*) *arthrostigma* (Lutz, 1905)	209 (17.7)
		*Wyeomyia* sp.	6 (0.5)
	Toxorhynchitini	*Toxorhynchites* (*Ankylorhynchus*) cf. *trichopygus* (Wiedemann, 1828)	10 (0.8)
		*Toxorhynchites* (*Lynchiella*) *bambusicola* (Lutz and Neiva, 1913)	10 (0.8)
**Total**			**1182 (100.0)**

**Fig 2:**
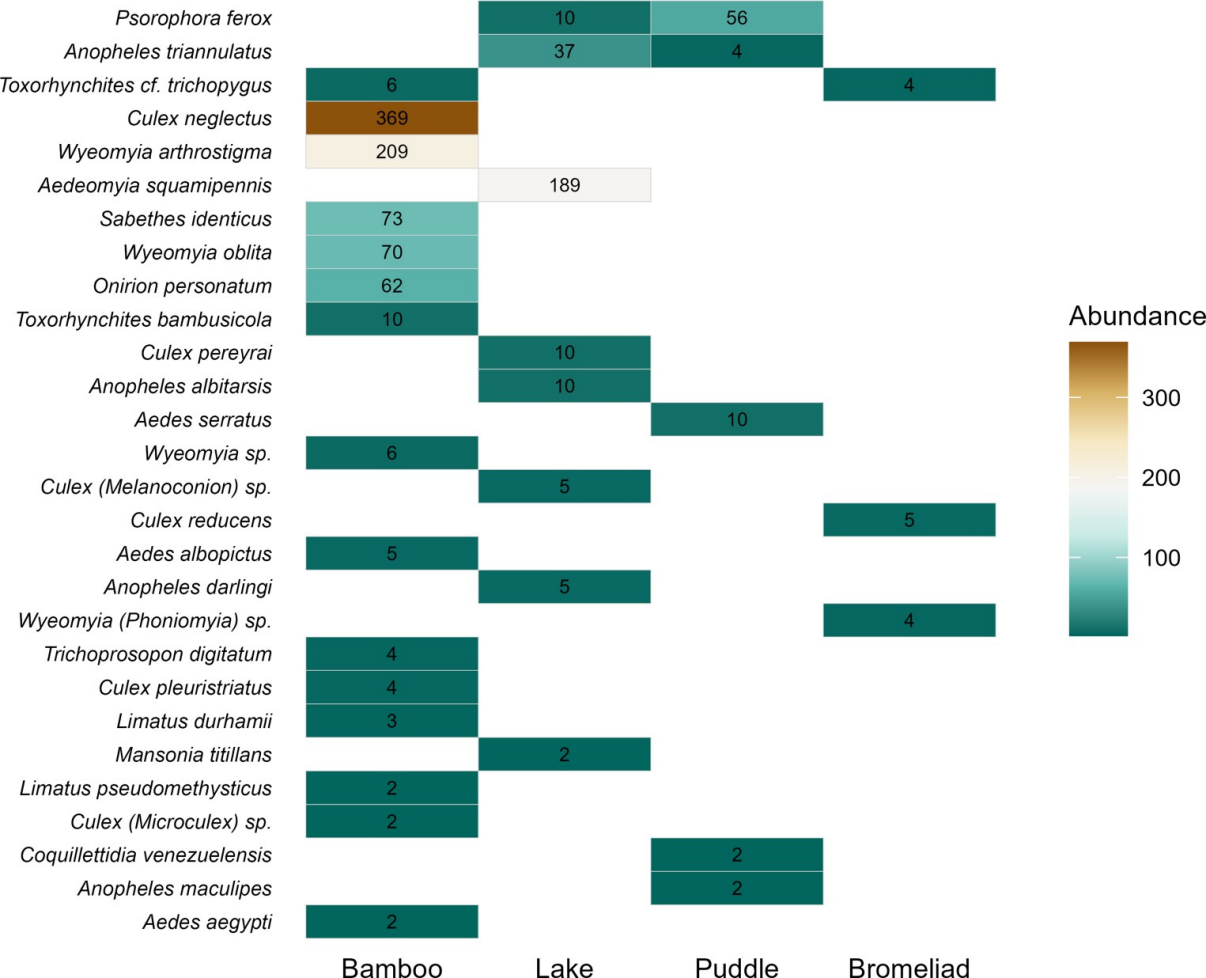
Species-by-site matrix for 28 mosquito species collected in four types of breeding sites in a forest fragment at the Poço das Antas Biological Reserve in southeastern Brazil. Rectangles represent species occurrence in each type of breeding site and are colored according to the number of individuals found therein. Breeding sites are ordered from left to right by the number of species, and species are ordered from top to bottom by the number of sites where the species occur, followed by the number of individuals. Note that only three species occur in more than one type of breeding habitat (*Psorophora ferox*, *Anopheles triannulatus*, and *Toxorhynchites* cf. *trichopygus*).

**Fig 3:**
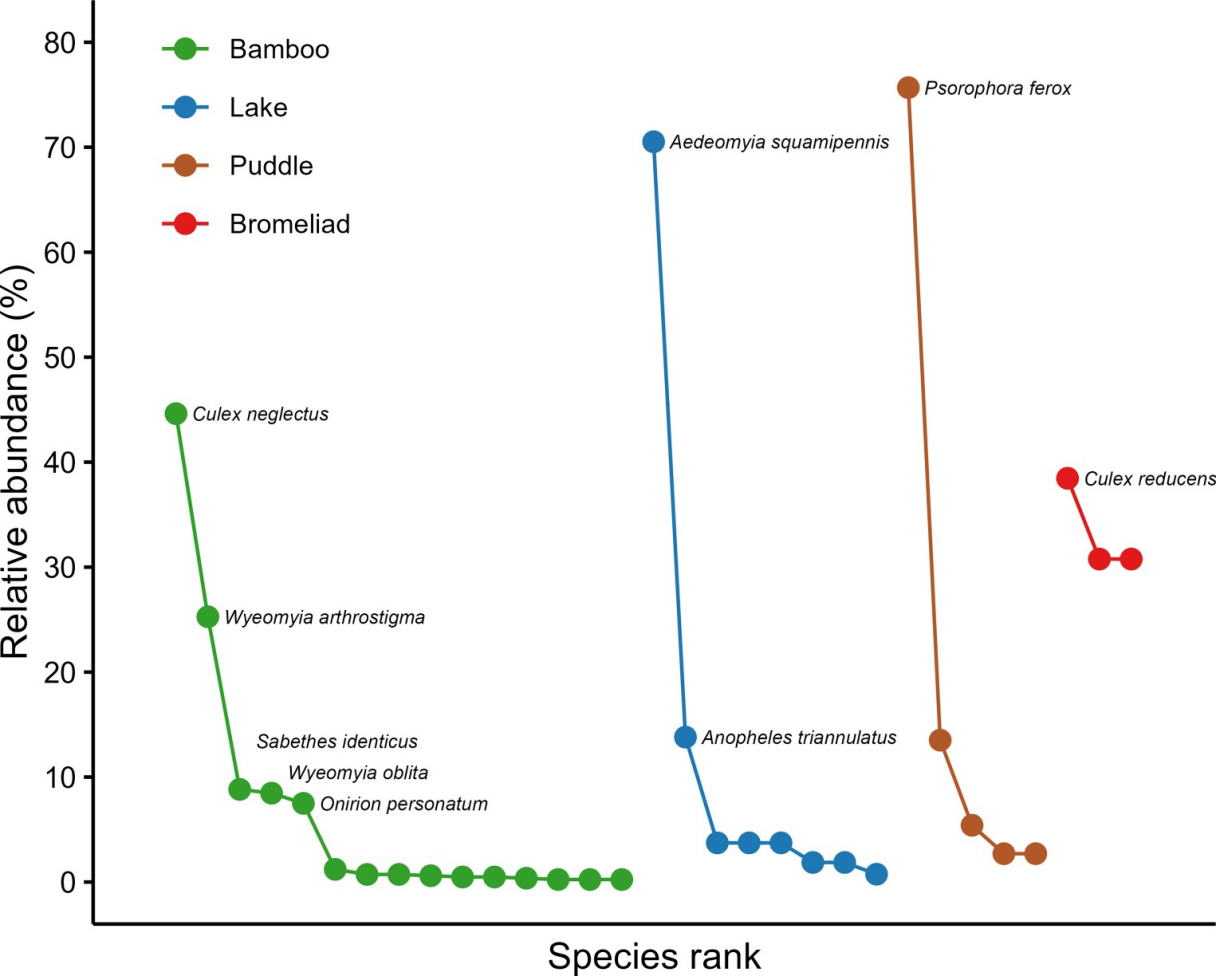
Relative abundance of mosquito species in four types of breeding sites in a forest fragment at the Poço das Antas Biological Reserve in southeastern Brazil. Points represent species, and colors represent the type of breeding habitat type. Dominant species–i.e., those whose relative abundance is higher than 1/*S*, where *S* is the number of species in a given breeding habitat–are indicated.

Mosquitoes were collected mainly in bamboos, with 827 individuals (69.9%) belonging to 15 species. In the lake, 268 individuals (22.7%) belonging to eight species were collected, while 74 specimens (6.3%) belonging to five species were collected in puddles. Only 13 individuals (1.1%) of three species were collected in bromeliads. Three species were found in more than one breeding site: *Ps*. *ferox* and *An*. *triannulatus* in the lake and puddle, and *Tx*. *trichopygus* in bamboos and bromeliads (Fig 2). Bamboo was the breeding ground with the highest number of dominant species (5), followed by the lake (2), puddle (1), and bromeliads (1) (Fig 3).

**Fig 4:**
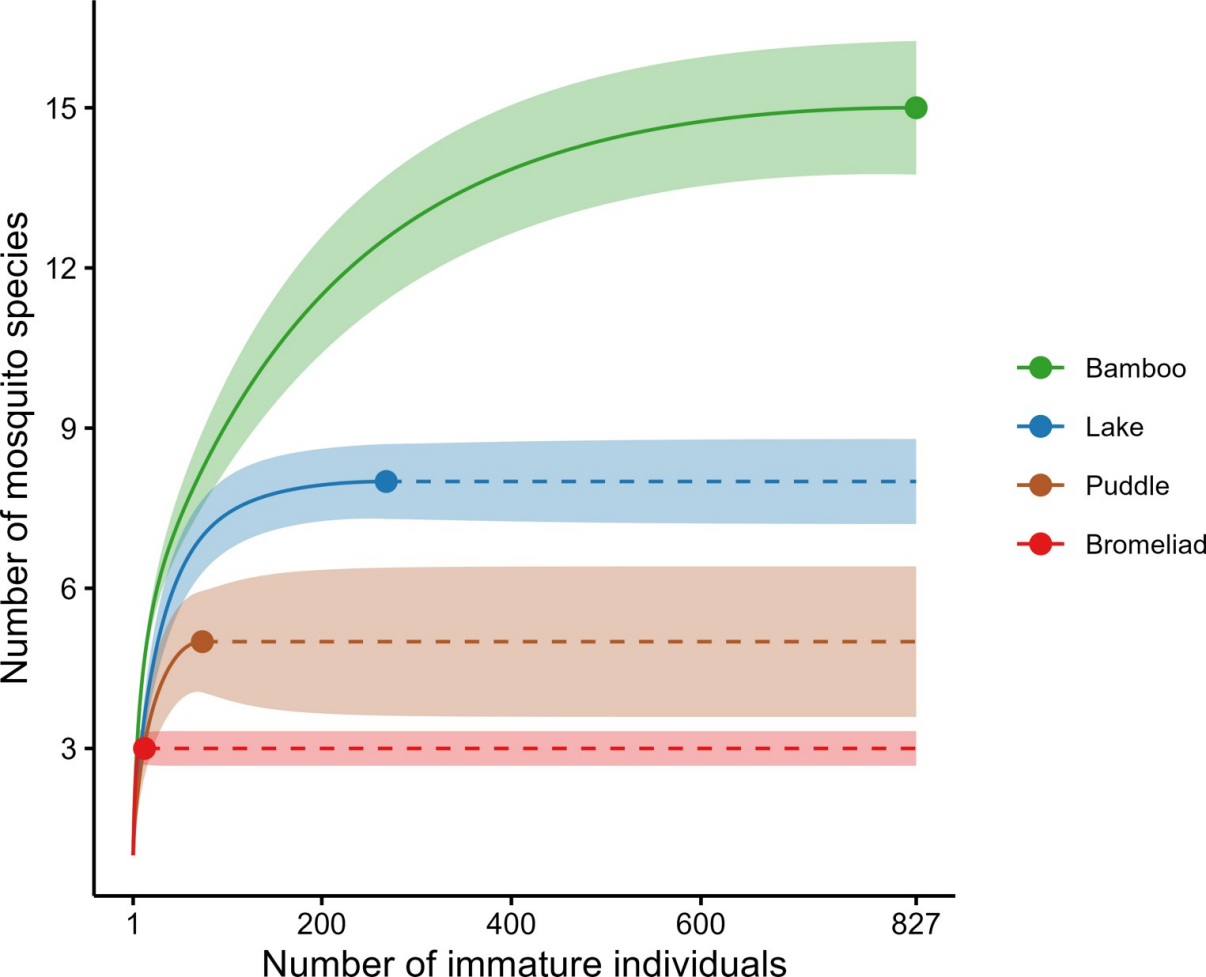
Individual-based species accumulation curves for mosquito species collected in four types of breeding sites in a forest fragment at the Poço das Antas Biological Reserve in southeastern Brazil. The curves are extrapolated to 827 individuals, which corresponds to the maximum number of individuals sampled among the four types of breeding sites. Solid lines, circles and dashed lines represent the interpolated, observed and extrapolated number of species, respectively. Shaded areas represent the 95% confidence intervals.

**Fig 5:**
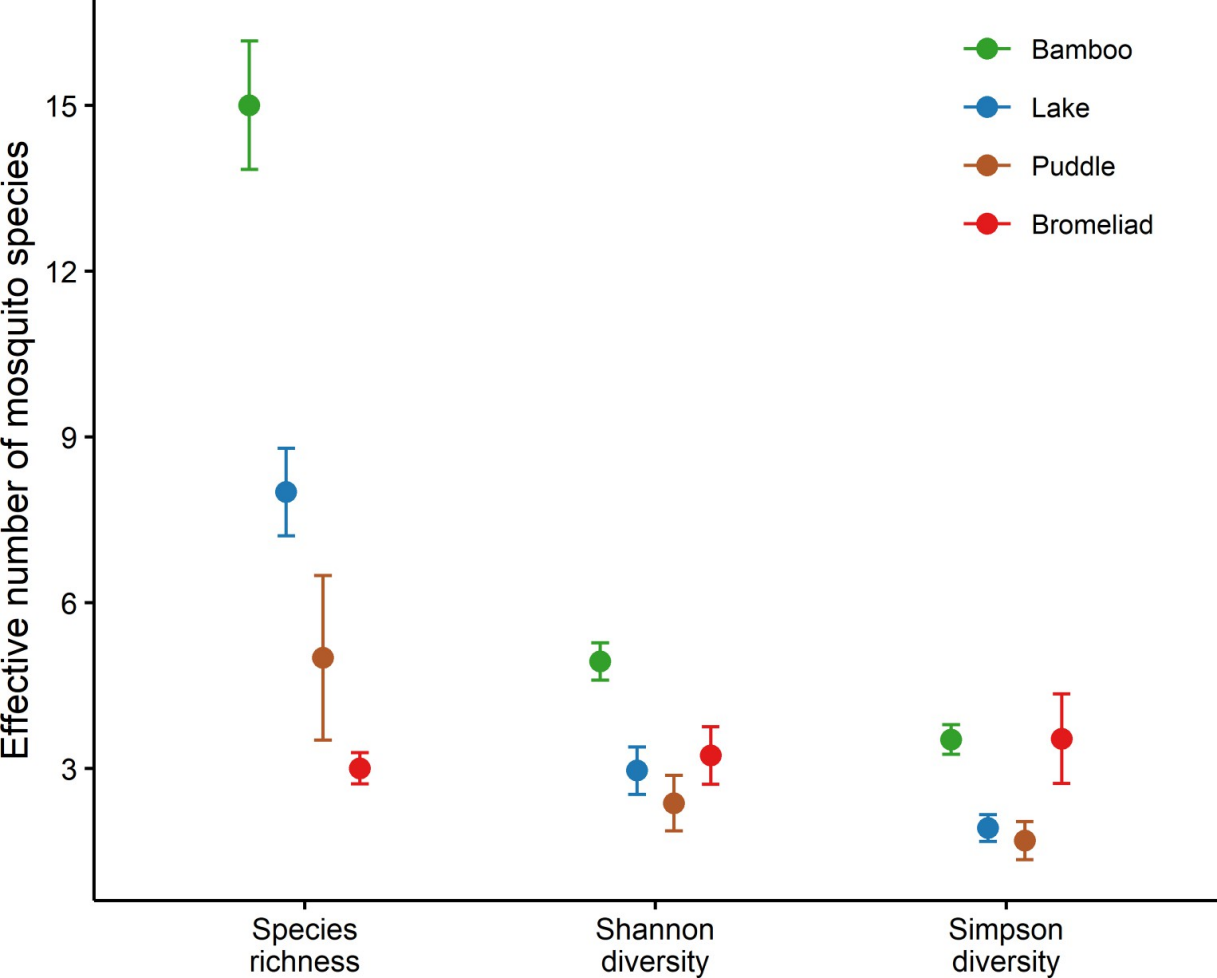
Diversity profile of mosquito assemblages found in four types of breeding sites in a forest fragment at the Poço das Antas Biological Reserve in southeastern Brazil. The graph shows the values of three diversity indices–(i) species richness, (ii) the exponential of Shannon’s entropy index (Shannon diversity), and (iii) the inverse of Simpson’s concentration index (Simpson diversity)–derived from extrapolation curves for 827 individuals (see Fig 4) for each type of breeding site. Error bars represent the 95% confidence intervals.

Individual-based species accumulation curves for the four types of breeding sites indicated that bamboos had the highest species richness, followed by the lake, puddle, and bromeliads (Fig 4). Despite significant differences in the species richness, the Shannon and Simpson diversity were broadly similar across the types of breeding sites. The former index indicated a higher number of typical species in the bamboos than in the three other types of breeding sites, which did not differ from each other. The latter index indicated a higher number of very abundant species in bamboos and bromeliads than in the lake and puddle (Fig 5).

## Discussion

We found 28 species of mosquitoes belonging to 13 genera, most of which have sylvatic habits, including the species from the tribes Sabethini and Toxorhynchitini and the subgenera of *Culex* (*Microculex*). However, taxa with habits associated with human environments, such as those from the tribes Mansoniini and Aedini, were also recorded. This apparent diversity in habit preference between the groups of collected mosquitoes may be associated with the composition of the environment at the PABioR. This strictly protected area is composed of a mosaic of second-growth forest in various stages of development and degrees of conservation, with vegetation covering about half of its area, with the rest consisting of pioneer formations and human-managed fields [33].

The PABioR was created is 1974 to protect populations of the globally endangered Golden Lion Tamarin (*Leontopithecus rosalia*) [34]. Despite this long history, studies on the mosquito fauna in the PABioR are scarce, especially in terms of the collection of immature forms. Alencar et al. [35] used ovitraps to obtain mosquito eggs in the reserve and recorded five species: *Ae*. *albopictus*, *Ae*. *terrens*, *Hg*. *leucocelaenus*, *Hg*. *capricornii*/*janthinomys* and *Cx*. *iridescense*. In another study involving the collection of eggs, Mello et al. [36] recorded a sixth species, *Ps*. *ferox*. However, none of these studies looked for immature forms of mosquitoes in their natural breeding sites. In a third mosquito survey at the PABioR, Alencar et al. [37] also collected only adult forms, finding 41 species from 12 genera. Of these, 14 species were recorded in the present study. Thus, 13 of the mosquito species recorded herein had not been previously reported in the PABioR, bringing the total number of species to 46.

PABioR is located in an area with recorded circulation of the yellow fever virus, with documented deaths of monkeys from yellow fever in 2018 [38]. In addition, dengue cases have been reported in the region containing this Biological Reserve, with more than one virus serotype appearing to circulate simultaneously [39]. *Haemagogus* species, which are vectors of the yellow fever virus in its sylvatic cycle, were not recorded in the present study since larvae were not collected in tree hollows, the natural breeding sites of these mosquitoes [40]. However, we did find *Ae*. *aegypti* and *Ae*. *albopictus* in samples from the internodes of perforated bamboos. *Aedes aegypti* is of high epidemiological importance since it transmits several arboviruses, including dengue and yellow fever viruses [41]. Meanwhile, although *Ae*. *albopictus* appears to play a role in spreading the dengue virus in rural areas of Asia, its epidemiological importance seems to be secondary in Brazil [42]. Although some specimens of *Ae*. *albopictus* from a wild area of Rio de Janeiro were found to be naturally infected with yellow fever and zika viruses, their role as vectors has not been established [43].

Of these two *Aedes* species, *Ae*. *albopictus* [44] has been recorded as using bamboos for breeding sites since it can breed both in perforated internodes [45] and in bamboo stumps [46]. *Aedes aegypti* seems to prefer only bamboo stumps for oviposition [47], which makes its occurrence in bamboo internodes quite unusual. Indeed, its presence in the samples is probably due to a strong human influence, given that the sampled bamboo grove is located on the edge of a small dirt road crossing part of the PABioR less than 1 km from the entrance of the Biological Reserve, which is in front of a main road. Moreover, Alencar et al. [48] observed populations of *Ae*. *aegypti* within areas of Atlantic Forest 700 m from the edge of the forest, demonstrating that these mosquitoes can invade forest environments.

With five specimens collected in the lake, *An*. *darlingi* is another species that merits attention due to its epidemiological importance. It is considered the primary malaria vector in Brazil and is distributed from the Amazon to the Atlantic Forest [49]. *Anopheles darlingi* can be found in natural and artificial breeding sites on the ground, preferably in ponds shaded by riparian vegetation [50]. Furthermore, the larvae of this species are notoriously difficult to collect and thus may not be found along with adult forms [51]. Hence, the reduced number of specimens collected in the present study can be explained by the natural difficulty of capturing immature forms of *An*. *darlingi* and the scarce riparian vegetation in the sampled lake area, which is widely exposed to solar radiation. It is important to note that prediction models indicate that global warming may displace the transmission of malaria by *An*. *darlingi* from the Amazon to the Atlantic Forest due to the plastic responses of the mosquito population to rising temperatures [52], justifying the constant monitoring of this species in PABioR and other wild areas of Rio de Janeiro.

The present results indicate that bamboos support a considerable part of the mosquito fauna in a wild environment of the Atlantic Forest, at least relative to the other breeding sites analyzed. We made holes in the sides of bamboo internodes using an electric drill, resembling holes made by larvae of Noctuidae (Lepidoptera) [53] and adults of Curculionidae (Coleoptera) [54], which occur in nature while the plant is still young. This procedure may have helped increase the production of mosquito larvae, as several potential breeding sites were artificially created in the same place, a departure from the pattern of natural drilling by these insects. Medeiros-Sousa et al. [55], studying Atlantic Forest fragments in urban parks of São Paulo, observed a lower richness of mosquitoes (three species) in bamboos naturally perforated by sylvatic insects than in natural breeding sites of other types, including bromeliads (six species) and ponds (four species).

We recorded 15 species of mosquitoes in bamboos, five of which were dominant: *Cx*. *neglectus*, *Wy*. *arthrostigma*, *Sa*. *identicus*, *Wy*. *oblita*, and *On*. *personatum*. This number of dominant species in bamboo was higher than the observed in the other analyzed breeding sites, resulting in a higher Simpson diversity, which was highest in bamboo and bromeliads. The Shannon diversity was also higher for bamboos than for the other breeding sites due to the larger number of typical species. The species richness observed by a study conducted in an Atlantic Forest fragment in Rio de Janeiro found 19 mosquito *taxa* in internodes of artificially perforated bamboos, including the five dominant species recorded herein, with *Cx*. *neglectus* as the most abundant too [56]. In another study carried out in an urban park of São Paulo, an area heavily impacted by human action, only seven *taxa* of mosquitoes inhabiting perforated bamboo internodes were observed, with *Wy*. *oblita* as the most abundant species [57].

Six specimens of *Toxorhynchites trichopygus* were collected from bamboos and four from bromeliads. This taxon was originally described as two species, *Ankylorhynchus neglectus* Lutz, 1904 and *Ankylorhynchus trichopygus* Dyar, 1928. The first species was described from larvae collected in bromeliads and the second from bamboo internodes. Subsequently, they were discovered to be synonymous and were later reclassified in the genus *Toxorhynchites* [58]. Some species of the genus *Toxorhynchites* follow the pattern of *Tx*. *trichopygus* regarding oviposition in breeding sites of different types, such as *Tx*. *amboinensis*, laying its eggs in artificial breeding sites, tree hollows, leaf axils, and bamboos [59].

We recorded another five species in bamboos at low abundance (≤ 10 specimens), *Tx*. *bamboosicola*, *Tr*. *digitatum*, *Cx*. *pleuristriatus*, *Li*. *durhamii*, and *Li*. *pseudomethysticus*. Most of these species had already been recorded in bamboos [56, 60], except for *Cx*. *pleuristriatus*, which had been recorded in bromeliads [61], and *Li*. *pseudomethysticus*, recorded in artificial breeding sites [62].

The lake was the second-richest type of breeding site of the four analyzed, and the dominant mosquito species there were *Ad*. *squamipennis* and *An*. (*Nys*.) *triannulatus*, and. *Aedeomyia squamipennis* has a Neotropical distribution, with abundant records in all Brazilian biomes [63]. This species is capable of being infected by several parasites participating in the cycle of the Gamboa virus [64], Equine Encephalitis virus [65], and *Plasmodium* spp., which causes avian malaria [66]. *Aedeomyia squamipennis* is directly associated with breeding sites containing aquatic plants [67], matching the sampling points on the lake. *Anopheles* (*Nyssorhynchus*) is capable of breeding in large permanent breeding sites formed in the ground by artificial and natural causes. In addition, the subgenus *Anopheles* (*Nyssorhynchus*) is the most common vector species of *Plasmodium* spp.

In contrast to the findings of Jules et al. [68] reported in a study of an area near PABioR, in which seven species of *An*. (*Nyssorhynchus*) were recorded, including *An*. *triannulatus*, we found only three species of this subgenus. As noted for *Ad*. *squamipennis*, the immature forms of *An*. *triannulatus* are commonly associated with breeding sites containing aquatic plants and grasses, as well as preferring water with ample light exposure [13, 69]. In addition to the lake, *An*. *triannulatus* was recorded in the sampled puddle, demonstrating a preference for various breeding sites formed from water accumulation in the soil. The puddle sample area was less than 0.5 m in depth with abundant herbaceous vegetation. A study carried out in the Amazon region also recorded collections of *An*. *triannulatus* in lakes and puddles [70].

The mosquito species collected in the lake had a lower Simpson diversity than in bamboos and bromeliads. The Shannon diversity for the lake did not differ statistically from the values observed in the puddle and bromeliads. Many factors can influence the abundance and composition of the mosquito community in a breeding site like a lake, including the rainfall regime and the presence of competitors, predators, algae, and aquatic plants [71, 72]. The present results show a relatively low number of mosquito species in the lake, with only eight *taxa* captured, including only one species of *Mansonia* (*Ma*. *titillans*), a genus often dominant in breeding sites of this kind [73]. Meanwhile, Lopes et al. [74] collected 17 mosquito taxa from a lake of an Atlantic Forest fragment under strong anthropic influence. Despite the presence of aquatic plants in the sampled lake, we did not collect any larvae from the roots of these plants, as the present collections were limited to the margins as far as the dippers reached. This methodology could explain the low number of *Mansonia* specimens collected. In addition, in the study conducted by Lopes et al. [74], the sampling effort was extended beyond the shores of the lake, which increased the chances of collecting a greater number of species.

Other species recorded in the lake were *Cx*. *pereyrai*, *An*. *albitarsis*, and *Ps*. *ferox*. *Culex pereyrai* can also be found on riverbanks, where the current is slow [75]. *Anopheles albitarsis* is considered a generalist species in terms of breeding sites, which can even use transient breeding sites formed by the accumulation of rainwater, puddles, and permanent breeding sites such as lakes [76, 77]. Meanwhile, *Ps*. *ferox*, which was also found in puddles in the present study, commonly occurs in transient breeding sites on the ground [78].

The puddle had the third-highest species richness, with five species recorded. In addition, the Shannon and Simpson diversity were the lowest in the puddle. In a study by Medeiros-Sousa et al. [57] an urban park where mosquitoes were collected in different types of breeding sites, puddles were second in terms of species richness, only behind lakes. Some characteristics of this type of breeding site, including the type of vegetation (e.g., presence of grasses) and exposure to sunlight, can affect the mosquito fauna [79].

*Psorophora ferox* was considered the only dominant species in the sampled puddle. Verna [80] collected immatures in different types of breeding sites in the United States of America and found that *Ps*. *ferox* was also classified into the dominant species group among the 44 species of mosquitoes collected. This species has its population growth directly affected by the rainfall since, in these periods, transitory breeding sites are formed in the soil, which favors its proliferation [13]. The other species recorded in the puddle were *An*. *triannulatus*, which was also recorded in the lake, *Ae*. *serratus*, *Cq*. *venezuelensis*, and *An*. *maculipes*. All these species have already been recorded in breeding sites with characteristics similar to the present study [81–83].

Bromeliads had the lowest number of mosquitoes of any breeding site surveyed, with only three *taxa*. However, Simpson diversity was relatively high, and statistically the same as Shannon diversity and species richness, since the number of individuals per species was virtually the same (i.e. high evenness). Bromeliads are considered one of the main breeding sites for sylvatic mosquitoes in the Atlantic Forest, with more than 30 species breeding in the water accumulated in bromeliads’ leaf axils [84–86]. Accordingly, Bastos et al. [87] observed a mosquito species richness higher in bromeliads than in bamboos and lakes. The bromeliads sampled in the PABioR were exposed to the sun, with little plant coverage, which implied a low deposition of organic material (e.g., sheets that could fall from the trees and deposit in the bromeliad). This may have influenced the present results since lower amounts of organic matter in the breeding site could have negatively influenced the composition of the insect fauna [88]. In addition, we sampled a small number of bromeliads (11 plants), which may also explain the relatively low mosquito species richness.

Of the three *taxa* recorded in bromeliads, *Cx*. *reducens* was the most abundants. This species belongs to the subgenus *Cx*. (*Microculex*), which frequently occurs in phytotelmata breeding sites, like *Wy*. (*Phoniomyia*) (e.g., Torreias et al. [89]). Moreover, *Cx*. *reducens* seems to be habitat generalist, as it is also found in bromeliads in urban and peri-urban areas [90].

Understanding the epidemiological context of the circulation of potentially pathogenic agents to humans that can be transmitted by mosquitoes is related to a greater understanding of the importance of each of the various types of breeding sites in the control of this fauna. The present results reinforce the need to monitor mosquito breeding sites in natural areas of the Atlantic Forest, especially those formed by bamboo internodes and lakes, due to the epidemiological importance of some species found, mainly *Ae*. *aegypti*, *Ae*. *albopictus* and *An*. *darlingi*. Particularly, we stress the importance of bamboos for maintaining a high number of sylvatic species of mosquitoes in the biome. Despite this, breeding sites formed by bamboos are neglected in vector control strategies, which can thereby pose risks to human populations living in settlements close to fragments of the Atlantic Forest. Since bamboos proved to be important for the maintenance of the mosquito fauna, we suggest their inclusion in actions to reduce breeding sites for vector control in places close to wild areas of the Atlantic Forest.

## Supporting information

S1 Fig Photograph of the four types of breeding sites sampled at the Poço das Antas Biological Reserve in southeastern Brazil. A and B: lake; C: bromeliad; D: puddle; E and F: bamboo.
(TIF)

S1 Table Data from the mosquito collections carried out at the Poço das Antas Biological Reserve in southeastern Brazil. (XLS)
