# Supplementary figures and images for: Little noticed, but very important: The role of breeding sites formed by bamboos in maintaining the diversity of mosquitoes (Diptera: Culicidae) in the Atlantic Forest biome
Source: PLoS One. 2022 Sep 6;17(9):e0273774. doi: 10.1371/journal.pone.0273774 (PMC9447929; doi:10.1371/journal.pone.0273774)

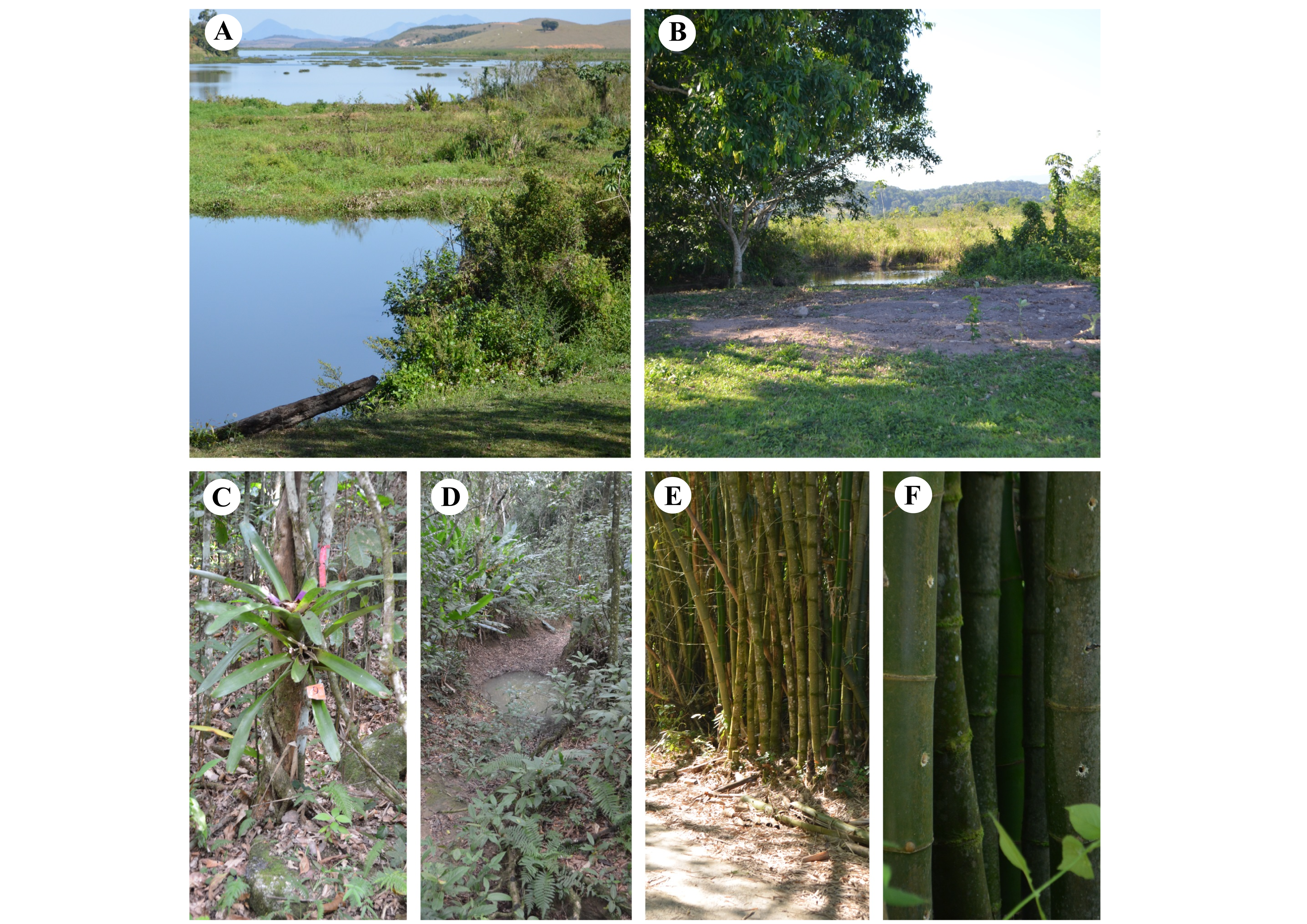

Supplement: S1 Fig — A and B: lake; C: bromeliad; D: puddle; E and F: bamboo. (TIF) [file pone.0273774.s001.tif]
